# The relationship between islet autoantibody status and the genetic risk of type 1 diabetes in adult-onset type 1 diabetes

**DOI:** 10.1007/s00125-022-05823-1

**Published:** 2022-11-10

**Authors:** Nicholas J. Thomas, Helen C. Walkey, Akaal Kaur, Shivani Misra, Nick S. Oliver, Kevin Colclough, Michael N. Weedon, Desmond G. Johnston, Andrew T. Hattersley, Kashyap A. Patel

**Affiliations:** 1 Institute of Biomedical and Clinical Science, University of Exeter Medical School, Exeter, UK; 2 Department of Diabetes and Endocrinology, Royal Devon and Exeter NHS Foundation Trust, Exeter, UK; 3 Faculty of Medicine, Imperial College London, London, UK

**Keywords:** Adult onset, Diabetes classification, Islet autoantibodies, Type 1 diabetes, Type 2 diabetes

## Abstract

**Aims/hypothesis:**

The reason for the observed lower rate of islet autoantibody positivity in clinician-diagnosed adult-onset vs childhood-onset type 1 diabetes is not known. We aimed to explore this by assessing the genetic risk of type 1 diabetes in autoantibody-negative and -positive children and adults.

**Methods:**

We analysed GAD autoantibodies, insulinoma-2 antigen autoantibodies and zinc transporter-8 autoantibodies (ZnT8A) and measured type 1 diabetes genetic risk by genotyping 30 type 1 diabetes-associated variants at diagnosis in 1814 individuals with clinician-diagnosed type 1 diabetes (1112 adult-onset, 702 childhood-onset). We compared the overall type 1 diabetes genetic risk score (T1DGRS) and non-HLA and HLA (DR3-DQ2, DR4-DQ8 and DR15-DQ6) components with autoantibody status in those with adult-onset and childhood-onset diabetes. We also measured the T1DGRS in 1924 individuals with type 2 diabetes from the Wellcome Trust Case Control Consortium to represent non-autoimmune diabetes control participants.

**Results:**

The T1DGRS was similar in autoantibody-negative and autoantibody-positive clinician-diagnosed childhood-onset type 1 diabetes (mean [SD] 0.274 [0.034] vs 0.277 [0.026], *p*=0.4). In contrast, the T1DGRS in autoantibody-negative adult-onset type 1 diabetes was lower than that in autoantibody-positive adult-onset type 1 diabetes (mean [SD] 0.243 [0.036] vs 0.271 [0.026], *p*<0.0001) but higher than that in type 2 diabetes (mean [SD] 0.229 [0.034], *p*<0.0001). Autoantibody-negative adults were more likely to have the more protective HLA DR15-DQ6 genotype (15% vs 3%, *p*<0.0001), were less likely to have the high-risk HLA DR3-DQ2/DR4-DQ8 genotype (6% vs 19%, *p*<0.0001) and had a lower non-HLA T1DGRS (*p<*0.0001) than autoantibody-positive adults. In contrast to children, autoantibody-negative adults were more likely to be male (75% vs 59%), had a higher BMI (27 vs 24 kg/m^2^) and were less likely to have other autoimmune conditions (2% vs 10%) than autoantibody-positive adults (all *p*<0.0001). In both adults and children, type 1 diabetes genetic risk was unaffected by the number of autoantibodies (*p>*0.3). These findings, along with the identification of seven misclassified adults with monogenic diabetes among autoantibody-negative adults and the results of a sensitivity analysis with and without measurement of ZnT8A, suggest that the intermediate type 1 diabetes genetic risk in autoantibody-negative adults is more likely to be explained by the inclusion of misclassified non-autoimmune diabetes (estimated to represent 67% of all antibody-negative adults, 95% CI 61%, 73%) than by the presence of unmeasured autoantibodies or by a discrete form of diabetes. When these estimated individuals with non-autoimmune diabetes were adjusted for, the prevalence of autoantibody positivity in adult-onset type 1 diabetes was similar to that in children (93% vs 91%, *p*=0.4).

**Conclusions/interpretation:**

The inclusion of non-autoimmune diabetes is the most likely explanation for the observed lower rate of autoantibody positivity in clinician-diagnosed adult-onset type 1 diabetes. Our data support the utility of islet autoantibody measurement in clinician-suspected adult-onset type 1 diabetes in routine clinical practice.

**Graphical abstract:**

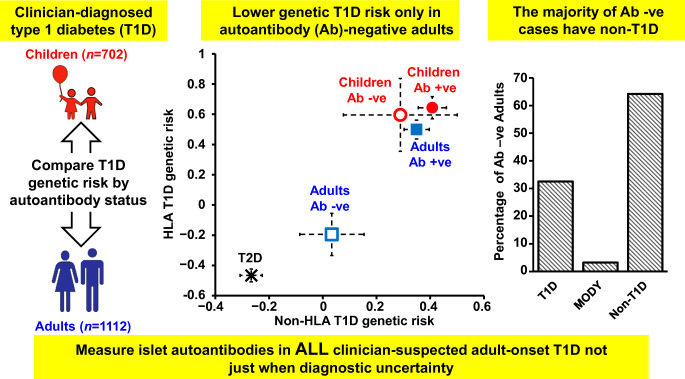

**Supplementary Information:**

The online version contains peer-reviewed but unedited supplementary material available at 10.1007/s00125-022-05823-1.

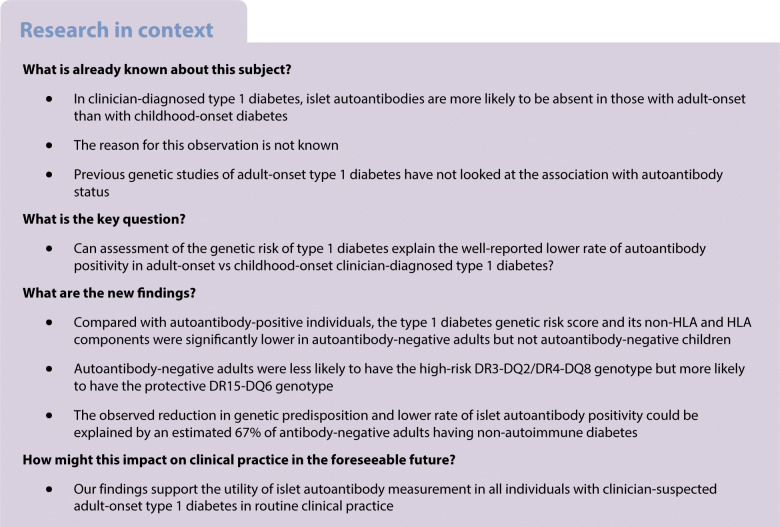



## Introduction

Our understanding of adult-onset type 1 diabetes is lagging behind that of childhood-onset type 1 diabetes. Type 1 diabetes is an autoimmune disease with a strong polygenic susceptibility that can present at all ages. In total, 40% of individuals present after the age of 30 years [1] and it is increasingly recognised that these individuals with adult-onset type 1 diabetes are immunologically different from those diagnosed in childhood. In childhood-onset type 1 diabetes, islet autoantibodies are present in ~90% of individuals at diagnosis [2–5], whereas, in clinician-diagnosed adult-onset type 1 diabetes, autoantibody positivity is reduced to approximately 70% [2, 6–9]. The reasons for this observed reduction are not known but it could be due to the presence of other unmeasured autoantibodies or a new discrete form of diabetes or the inadvertent misclassification of non-autoimmune diabetes. Genetic predisposition to type 1 diabetes has been shown to influence the presence of autoantibodies [10–14]. Therefore, detailed immunogenetic studies may help us to better understand the reasons for the lower prevalence of autoantibody positivity in adult-onset type 1 diabetes studies and improve our understanding of the pathophysiology and clinical utility of autoantibodies in adult-onset type 1 diabetes.

The genetic risk of type 1 diabetes has been well studied in European populations and multiple HLA and non-HLA variants associated with type 1 diabetes have been identified [15, 16]. These variants can be combined into a type 1 diabetes genetic risk score (T1DGRS) providing the overall genetic predisposition to type 1 diabetes in an individual. The T1DGRS is useful for discriminating type 1 diabetes from non-autoimmune diabetes (including monogenic and type 2 diabetes) but is not routinely used by clinicians to diagnose type 1 diabetes [17, 18], providing an unbiased opportunity to assess the association of type 1 diabetes genetic predisposition with autoantibody status in adults. The previous genetic studies of adult-onset type 1 diabetes have looked at the genetic risk of type 1 diabetes in either autoantibody-positive individuals or autoantibody-negative and -positive individuals together [19, 20]. In this study, we aimed to assess the genetic risk of type 1 diabetes based on 30 known type 1 diabetes-associated loci by autoantibody status in adult-onset clinician-diagnosed type 1 diabetes compared with childhood-onset type 1 diabetes to evaluate the reported lower prevalence of autoantibody positivity in adult-onset type 1 diabetes.

## Methods

### Study population

Participants with clinician-diagnosed type 1 diabetes were recruited to the UK-wide After Diabetes Diagnosis REsearch Support System-2 (ADDRESS-2) study. The detailed study protocol has been published elsewhere [21]. Participants were aged over 4 years at recruitment, insulin treated from diagnosis and recruited within 6 months of diagnosis. As there are no set criteria for diagnosis of type 1 diabetes, the diagnosis was based solely on the judgement of the treating clinician [22]. Participants recruited up to September 2017 were included in the present analysis. DNA and serum samples were collected alongside clinical characteristics and symptoms at diagnosis. Further details of participant characterisation is provided in the electronic supplementary material (ESM).

We studied those who self-reported as being White European (*n*=1866), the ethnicity for which genetic risk of type 1 diabetes is well studied. Participants were excluded if they were missing autoantibody data (*n*=29) or genetic data (*n*=23) (ESM Fig 1). The majority of individuals were hospitalised at diagnosis (74%), had severe hyperglycaemia (mean HbA_1c_ 87 mmol/mol [10.1%]) and were symptomatic (95% reported polyuria or polydipsia, 85% reported weight loss and 41% presented in diabetic ketoacidosis) (ESM Table 1). In total, 99% (1798/1814) of participants were hospitalised at diagnosis, had symptoms consistent with severe hyperglycaemia or both. Because of the widely reported reduction in T1DGRS with age [6, 19, 23], we conducted all analyses separately for children (<18 years at diagnosis) and adults (≥18 years at diagnosis).

### Islet autoantibody analysis

GAD autoantibodies (GADA), insulinoma-2 antigen autoantibodies (IA2A) and zinc transporter-8 autoantibodies (ZnT8A) were measured by the University of Bristol using established radiobinding assays [24, 25]. Islet autoantibodies were considered positive if the GADA titre was ≥97th centile of 974 non-diabetic control participants, the IA2A titre was ≥98th centile of 500 non-diabetic control participants and the ZnT8A titre was ≥97.5th centile of 523 non-diabetic control participants [2]. In the 2015 Islet Autoantibody Standardization Program Workshop, the assay sensitivities and specificities achieved were 74% and 96.7% for GADA, 72% and 100% for IA2A, 60% and 100% for ZnT8RA (arginine) and 46% and 100% for ZnT8WA (tryptophan), respectively.

### Genetic analysis

Details of T1DGRS generation, HLA genotyping and monogenic diabetes testing have been published elsewhere [17, 18, 26, 27] and are provided in the ESM.

### Statistical analysis

The χ^2^ test was used to compare categorical variables between the groups separated by age and autoantibody status and Student’s *t* test was used to compare continuous variables. Where there were more than two groups ANOVA was used to compare categorical variables and linear regression was used to compare continuous variables. We used the Wald binomial CIs method throughout to calculate 95% CIs for proportions. For analysis, non-HLA and HLA T1DGRS were scaled to have a mean of 0 and SD of 1. We performed sensitivity analysis by splitting the adult-onset diabetes group by the median age of the cohort (30.95 years). In those aged 18 years or younger at recruitment, BMI was age adjusted to adult levels using UK WHO (2007) reference data [28]. All analyses were performed using Stata 16 (StataCorp LP, College Station, TX, USA).

### Estimating the proportions of type 1 diabetes and non-autoimmune diabetes among the autoantibody-negative group

To estimate the proportions of individuals with type 1 diabetes and non-autoimmune diabetes among the autoantibody-negative group, we used the mean T1DGRS for autoantibody-positive type 1 diabetes (representing type 1 diabetes, clinical suspicion with autoantibody positivity) and for type 2 diabetes participants from the Wellcome Trust Case Control Consortium (representing non-autoimmune diabetes; see the ESM for further details) [29]. We then used the following formula to calculate the relative proportions of type 1 diabetes and non-autoimmune diabetes needed to achieve the mean T1DGRS observed in the autoantibody-negative group:

$$ \mathrm{Estimated}\ \mathrm{proportion}\ \mathrm{with}\ \mathrm{T}1\mathrm{D}\ \mathrm{in}\ \mathrm{autoantibody}\hbox{-} \mathrm{negative}\ \mathrm{group}=\frac{{\mathrm{auto}\mathrm{antibody}\ \mathrm{negative}}^{\mathrm{mean}\ \mathrm{GRS}}-\mathrm{Non}\_\mathrm{T}1{\mathrm{D}}^{\mathrm{mean}\ \mathrm{GRS}}}{\mathrm{auto}{\mathrm{antibody}\ \mathrm{positive}}^{\mathrm{mean}\ \mathrm{GRS}}-\mathrm{Non}\_\mathrm{T}1{\mathrm{D}}^{\mathrm{mean}\ \mathrm{GRS}}} $$

$$ \mathrm{Estimated}\ \mathrm{proportion}\ \mathrm{of}\ \mathrm{non}\hbox{-} \mathrm{autoimmune}\ \mathrm{diabetes}=1\hbox{--} \mathrm{proportion}\ \mathrm{with}\ \mathrm{type}\ 1\ \mathrm{diabetes} $$

We performed a sensitivity analysis using only those positive for all three autoantibodies to represent type 1 diabetes to reduce the possibility of false-positive autoantibody cases. The advantage of this method is that it does not use a T1DGRS cut-off to estimate type 1 diabetes, as type 1 diabetes can develop in those with a low genetic predisposition [30]. Instead, it compares T1DGRS distributions at a group level and provides robust group-level estimates [1]. We recently published a detailed methodology paper and validation of this method that discusses in detail a number of cautions and assumptions associated with using genetic risk scores in this context [31].

### Ethics statement

Ethics approval for the ADDRESS-2 study was granted by the South Central – Berkshire NHS Research Ethics Committee on 3 October 2010 (ref: 10/H0505/85). Ethics approval for monogenic testing in the ADDRESS-2 cohort was granted by the East of England – Essex Research Ethics Committee on 14 July 2016 (ref. 16/EE/0306).

## Results

### Islet autoantibody positivity is lower in clinician-diagnosed adult-onset type 1 diabetes

The analysis of GADA, IA2A and ZnT8A at diagnosis showed that 91% of children were positive for one or more autoantibody compared with 80% of adults (*p*<0.0001) (ESM Table 1). The proportion of participants with autoantibody positivity decreased with increasing age, from 88% of those diagnosed aged 18–30.95 years (*n=*556, diagnosed at less than the median age of the adult group) to 73% in those diagnosed aged >30.95–75 years (*n=*556, diagnosed at more than the median age of the adult group) (*p<*0.0001) (ESM Table 2).

### The T1DGRS of autoantibody-negative adults but not children is intermediate between that of autoantibody-positive individuals and non-autoimmune diabetes control participants

**Fig. 1:**
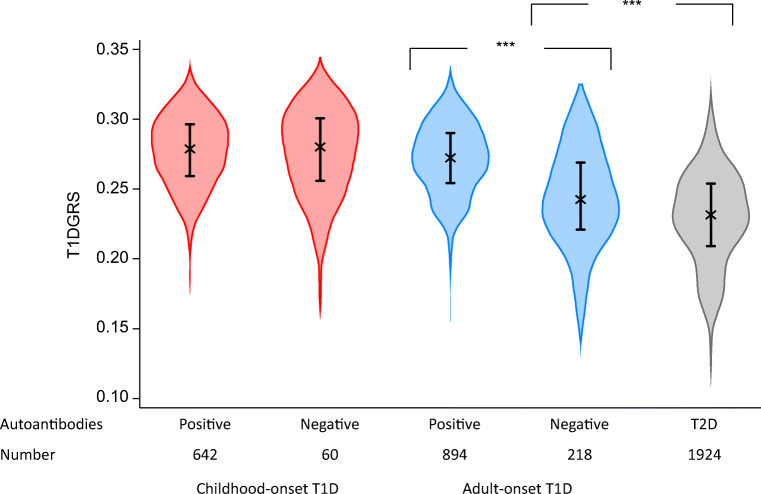
T1DGRS by islet autoantibody status in clinician-diagnosed adult- and childhood-onset type 1 diabetes (T1D). Individuals with type 2 diabetes (T2D) were from the Wellcome Trust Case Control Consortium [29]. Individuals with childhood-onset diabetes were those diagnosed at <18 years of age and individuals with adult-onset diabetes were those diagnosed at ≥18 years of age. ***p<0.001

We first analysed the T1DGRS in autoantibody-positive and -negative children and adults to gain an understanding of the lower prevalence of autoantibody positivity in adults. The T1DGRS was similar in autoantibody-negative and -positive children (mean [SD] 0.274 [0.034] vs 0.277 [0.026], *p=*0.4) (Fig. 1). However, the T1DGRS was significantly lower in autoantibody-negative adults than in autoantibody-positive adults (0.243 [0.036] vs 0.271 [0.026], difference 0.028 [95% CI 0.024, 0.032], *p*<0.0001) (Fig. 1 and ESM Table 3) but higher than that in non-autoimmune diabetes control participants (mean [SD] 0.229 [0.034], difference 0.014 [95% CI 0.009, 0.019], *p*<0.0001). The difference in T1DGRS between autoantibody-positive and autoantibody-negative adults increased further with older age at diagnosis (18–30.95 years: 0.019 [95% CI 0.012, 0.026]; >30.95 years: 0.032 [95% CI 0.026, 0.037]). Conversely, the difference in T1DGRS between autoantibody-negative adults and non-autoimmune diabetes control participants was reduced with older age at diagnosis (18–30.95 years: 0.023 [95% CI 0.015, 0.031]; >30.95 years: 0.009 [95% CI 0.004, 0.015]) (ESM Fig. 2 and ESM Table 4).

### Both HLA and non-HLA type 1 diabetes risks are lower in autoantibody-negative adults but not in children

**Fig. 2:**
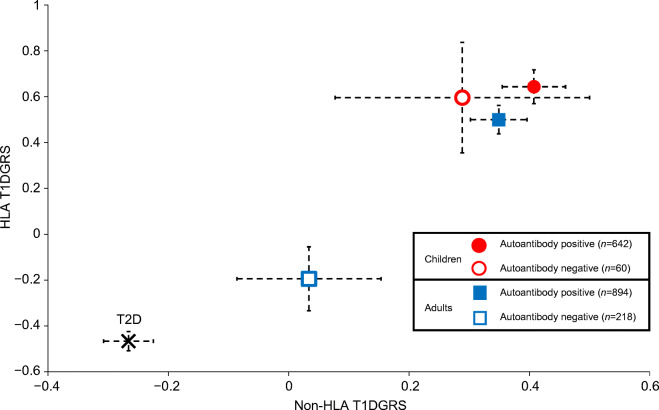
HLA and non-HLA T1DGRS by islet autoantibody status in clinician-diagnosed adult- and childhood-onset type 1 diabetes. Individuals with type 2 diabetes (T2D) were from the Wellcome Trust Case Control Consortium [29]. Non-HLA and HLA T1DGRS were scaled to have a mean of 0 and SD of 1 for ease of representation. Individuals with childhood-onset diabetes were those diagnosed at <18 years of age and individuals with adult-onset diabetes were those diagnosed at ≥18 years of age

**Fig. 3:**
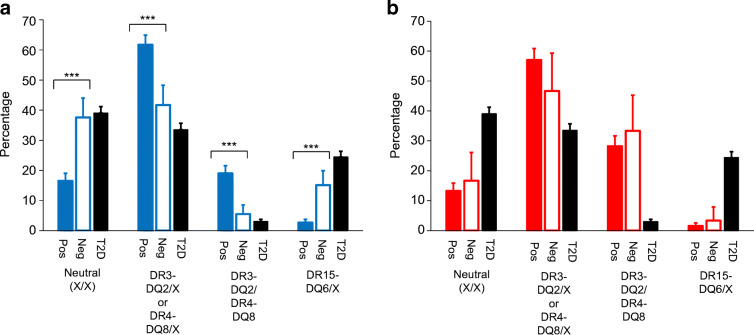
The frequencies of type 1 diabetes risk-increasing HLA genotypes and protective HLA genotypes by islet autoantibody status in clinician-diagnosed adult- and childhood-onset type 1 diabetes. (**a**) Age ≥18 years; (**b**) age <18 years. Neutral X/X represents any HLA other than DR3-DQ2, DR4-DQ8 or DR15-DQ6. DR3-DQ2/X or DR4-DQ8/X represents one of the following: DR3-DQ2/X, DR3-DQ2/DR3-DQ2, DR4-DQ8/X or DR4-DQ8/DR4-DQ8. DR15-DQ6/X represents DR15-DQ6/X or DR15-DQ6/DR15-DQ6. T2D, type 2 diabetes. ***p<0.001

We next compared the HLA and non-HLA components of the T1DGRS to assess which is driving the lower overall genetic risk in autoantibody-negative individuals. Both HLA and non-HLA components of the T1DGRS were lower in autoantibody-negative adults than in autoantibody-positive adults (both *p*<0.0001) but higher than in non-autoimmune diabetes control participants (both *p*<0.0001) (Fig. 2 and ESM Table 3). In autoantibody-negative adults, the frequencies of high-risk HLA genotypes were lower than in autoantibody-positive adults (DR3-DQ2 [DRB1*03:01-DQA1*05:01-DQB1*02:01]/DR4-DQ8 [DRB1*04-DQA1*03-DQB1*03:02]: 6% vs 19%; DR3-DQ2 or DR4-DQ8 homozygous or heterozygous without DR15-DQ6 [DRB1*15-DQA1*01-DQB1*06]: 42% vs 62%; both *p*<0.0001 [Fig. 3a and ESM Table 3]), but higher than in non-autoimmune diabetes control participants. Autoantibody-negative adults were more likely than autoantibody-positive adults to have one or more copies of the protective DR15-DQ6 haplotype (15% vs 3%, *p<*0.0001 [Fig. 3a and ESM Table 3]). In contrast, no difference in HLA or non-HLA risk was noted between autoantibody-negative and autoantibody-positive children (all *p*>0.1) (Figs 2 and 3b and ESM Table 3).

These differences in HLA and non-HLA genetic risk between autoantibody-negative and autoantibody-positive adults became more marked with higher age at diagnosis (ESM Table 4). Analysis of the individual frequency of non-HLA variants by autoantibody status gave results that were directionally consistent with the overall T1DGRS (ESM Table 5).

### Unmeasured or unknown autoantibodies may not explain the lower T1DGRS in autoantibody-negative adults

There are contrasting possible explanations for the unexpected finding of a lower genetic risk of type 1 diabetes in autoantibody-negative adults. The lower observed positive autoantibody prevalence could be explained by the presence of either unmeasured or unknown autoantibodies in individuals with adult-onset diabetes. If this is the case, measuring these autoantibodies would remove the observed difference in T1DGRS between autoantibody-positive and autoantibody-negative adults. To test this, we analysed data in adults with and without ZnT8A measurements (the most recently discovered autoantibody of those tested) to assess the impact of measuring additional autoantibodies over GADA and IA2A. We found that ZnT8A measurement additionally identified a small number of autoantibody-positive adults (*n=*14), corresponding to only 6% (14/232) of the adults negative for both GAD and IA2A. These 14 adults had a higher T1DGRS than the adults who were negative for all three autoantibodies (0.272 vs 0.243, *p*<0.01) (ESM Fig. 3). These data together suggest that additional measurement of unknown or unmeasured autoantibodies will not increase the observed T1DGRS of the autoantibody-negative group.

### Clinical features of autoantibody-negative adults are different from those of autoantibody-positive adults

If the majority of autoantibody-negative adults have autoimmune type 1 diabetes and are falsely negative for autoantibodies, we would expect the clinical features of autoantibody-negative individuals to be similar to those of autoantibody-positive individuals. In line with this hypothesis, there was no meaningful difference in clinical features at diagnosis between autoantibody-negative and autoantibody-positive childhood-onset cases (ESM Table 6). In contrast, autoantibody-negative adults were more likely than autoantibody-positive adults to be male (75% vs 59%) and have a higher BMI (27 kg/m^2^ vs 24 kg/m^2^) and were far less likely to have a pre-existing autoimmune condition (2% vs 10%; all *p*<0.0001; Table 1). Autoantibody-negative adults were also more likely to have a parent with diabetes (33% vs 19%, *p*<0.0001) despite the lower T1DGRS observed. These differences between autoantibody-negative and autoantibody-positive adults were largely more pronounced with higher age at diagnosis (ESM Table 4).

**Table 1:** Clinical features of clinician-diagnosed adult-onset type 1 diabetes by autoantibody status

Clinical feature	Autoantibody positive (*n*=894)	Autoantibody negative (*n*=218)	*p* value
At diagnosis			
Age at diagnosis (years)	32 (11)	38 (12)	<0.0001
Sex			
Male	524 (59)	164 (75)	<0.0001
Female	370 (41)	54 (25)	<0.0001
Diabetic ketoacidosis	377 (42)	83 (38)	0.3
Weight loss	773 (86)	186 (85)	0.7
Polyuria or polydipsia	839 (94)	199 (91)	0.2
Hospitalised at admission	559 (63)	130 (60)	0.4
At recruitment			
Diabetes duration (weeks)	11 (7)	11 (7)	0.7
HbA_1c_ (mmol/mol)	91 (32)	100 (36)	<0.001
HbA_1c_ (%)	10.5 (5.1)	11.3 (5.4)	<0.001
BMI (kg/m^2^)	24 (5)	27 (7)	<0.0001
On concurrent oral glucose-lowering agent	41 (5)	23 (11)	<0.01
Another autoimmune disease	92 (10)	4 (2)	<0.0001
Parent with diabetes	171 (19)	72 (33)	<0.0001

Data are mean (SD) or *n* (%)

### A discrete form of diabetes in all autoantibody-negative adults may not completely explain the observed results

The differences in clinical features between autoantibody-negative and autoantibody-positive adults along with the lower genetic risk of type 1 diabetes raises the possibility that there is a discrete form of diabetes with a different immunogenetic signature and different clinical features in autoantibody-negative adults. If the autoantibody-negative group represents a discrete form of diabetes (a lower T1DGRS with no autoantibodies), one might expect the T1DGRS to decrease with a decreasing number of positive autoantibodies, and that individuals with the lowest T1DGRS would be more likely to be autoantibody negative. However, in both adults and children, the HLA T1DGRS and non-HLA T1DGRS, as well as the clinical features, were similar, irrespective of being positive for one, two or three autoantibodies (ESM Table 7 and ESM Figs 4 and 5). The prevalence of autoantibody positivity was also similar across the quartiles of T1DGRS in children (11% in lowest quartile of T1DGRS vs 11% in highest quartile, *p*=0.99; data not shown). These data suggest that if there is a discrete form of diabetes it has a distinct genetic association, only occurs in the absence of autoantibodies and is not seen in children but becomes increasingly common with higher age at diagnosis in adults.

### Clinical features differ by T1DGRS in autoantibody-negative adults but not autoantibody-positive adults

We next explored the impact of T1DGRS on clinical features in autoantibody-negative adults, hypothesising that if there is one discrete form of diabetes the clinical phenotype should be homogeneous irrespective of genetic predisposition to type 1 diabetes. We analysed the clinical features of autoantibody-negative and autoantibody-positive adults split by the median T1DGRS of all adults in the cohort. In autoantibody-positive adults there was no difference in clinical features between those with a lower and a higher T1DGRS (ESM Table 8). Conversely, in autoantibody-negative adults, those with a lower T1DGRS had a higher BMI (27 kg/m^2^ vs 25 kg/m^2^, *p*=0.01) and were older at diagnosis (39 years vs 34 years, *p*=0.02) (ESM Table 8). These data support the hypothesis that the autoantibody-negative adult group is heterogeneous, including the presence of adults with non-autoimmune diabetes.

### Non-autoimmune diabetes in 67% of autoantibody-negative adults may explain the observed intermediate T1DGRS

A final potential explanation for the observed difference in clinical features and the intermediate T1DGRS in the autoantibody-negative group is that it represents a mixture of type 1 diabetes (false-negative autoantibodies with a genetic predisposition to type 1 diabetes similar to that in autoantibody-positive individuals) and some proportion of individuals with non-autoimmune diabetes (e.g. monogenic or type 2 diabetes; known to have a lower type 1 diabetes genetic risk and not associated with autoantibodies). Using the mean T1DGRS of the autoantibody-negative group (possible combination of type 1 diabetes and non-autoimmune diabetes), autoantibody-positive group (representing type 1 diabetes diagnoses based on clinical suspicion with autoantibody positivity) and type 2 diabetes participants from the Wellcome Trust Case Control Consortium (representing non-autoimmune diabetes), we estimated that the observed reduction in T1DGRS in autoantibody-negative adults could be explained by the inadvertent inclusion of 147/218 (95% CI 133, 160) individuals with non-autoimmune diabetes, with 71/218 (95% CI 58, 85) individuals having type 1 diabetes. Thus, individuals with non-autoimmune diabetes can be estimated to represent 67% (95% CI 61%, 73%) of all autoantibody-negative adults and 13% (95% CI 11%, 15%) of all 1112 adults with clinician-diagnosed type 1 diabetes. The estimated prevalence of non-autoimmune diabetes was even higher with later age of onset (77% [95% CI 71%, 84%] in those aged >30.95 years vs 45% [95% CI 34%, 57%] in those aged 18–30.95 years). A sensitivity analysis was performed defining adult type 1 diabetes by triple autoantibody positivity (to exclude the impact of any inadvertent false autoantibody-positive individuals). This provided a very similar estimate of 69% (95% CI 62%, 75%) of autoantibody-negative individuals having non-autoimmune diabetes.

### Some autoantibody-negative adults have monogenic diabetes

Given the possibility of non-autoimmune diabetes in autoantibody-negative adults, we conducted genetic testing for monogenic diabetes (a rare cause of non-autoimmune diabetes) in all 218 autoantibody-negative adults. We identified seven adults with monogenic diabetes (3% [95% CI 1%, 7%]; two with *HNF1B*-related diabetes and five with the mitochondrial DNA mutation m.3243A>G). Adults with monogenic diabetes had a lower mean (SD) T1DGRS than autoantibody-positive adults (0.218 [0.025] vs 0.271 [0.026], *p*<00001) but this did not explain the majority of lower type 1 diabetes risk in the autoantibody-negative group (mean [SD] T1DGRS: 0.243 [0.036] in the whole autoantibody-negative group vs 0.244 [0.036] in antibody-negative individuals without monogenic diabetes, *p*=0.8). This suggests that, if our finding does result from the inadvertent inclusion of non-autoimmune diabetes in 67% of autoantibody-negative adults, the majority are most likely to have atypical type 2 diabetes (the most common cause of non-autoimmune diabetes in adults). Group characteristics of the adults with monogenic diabetes are given in ESM Table 9.

### Prevalence of positive autoantibodies at diagnosis is similar in adults and children after exclusion of individuals estimated to have non-autoimmune diabetes

The observed prevalence of positive autoantibodies of 80% (894/1112) in the adult-onset group (ESM Table 1) was increased to 93% (894/965, 95% CI 91%, 94%) after excluding those estimated to have non-autoimmune diabetes (*p<*0.0001). The prevalence remained the same even at higher age of diagnosis (92% [95% CI 90%, 95%] in those aged >30.95 years and 93% [95% CI 91%, 95%] in those aged 18–30.95 years) and was similar to that in individuals with childhood-onset diabetes (642/702, 91% [95% CI 89%, 94%], *p*=0.4). In adult autoantibody-positive individuals, GADA was most common and was present in 90% (805/894) of individuals.

## Discussion

Our assessment of the genetic risk for type 1 diabetes in a large cohort of individuals with clinician-diagnosed type 1 diabetes shows that, in contrast to children, autoantibody-negative adults have an intermediate genetic predisposition to type 1 diabetes that becomes more marked with increasing age at onset. The lower T1DGRS in autoantibody-negative adults resulted from a reduction in both HLA and non-HLA risk, with a marked increase in DR15-DQ6 (protective against type 1 diabetes) and reduction in DR3-DQ2 or DR4-DQ8 associated with type 1 diabetes risk. Within the limits of the study, we explored possible explanations and propose that the inadvertent inclusion of individuals with non-autoimmune diabetes may explain this observation and thus the lower observed autoantibody positivity in adult-onset clinician-diagnosed type 1 diabetes.

Non-autoimmune diabetes is not associated with either autoantibodies [32] or genetic risk for type 1 diabetes [17, 18]. Any individuals with non-autoimmune diabetes would be included in the autoantibody-negative group, leading to an intermediate genetic risk of type 1 diabetes, as we observed in our study. Type 1 diabetes can occur with a low genetic risk and the absence of autoantibodies, meaning that, although having a lower genetic risk of type 1 diabetes reduces the chance of having autoimmune diabetes, it does not exclude autoimmune diabetes completely [17, 18]. After excluding the group estimated to have non-autoimmune diabetes, 93% of adult-onset type 1 diabetes participants had positive antibodies, which was comparable to the rate in childhood-onset type 1 diabetes participants. This result mirrors our previous study that used a method independent of clinical features to define type 1 diabetes (insulin deficiency), showing comparable proportions of positive autoantibodies across age groups, although the previous study measured antibodies many years after diagnosis [33]. Our finding that GADA is the most common autoantibody in adult-onset type 1 diabetes has been observed before, suggesting similarities between our study and previous studies of adult-onset type 1 diabetes [2, 7]. In autoantibody-positive adults, the similarity of the T1DGRS regardless of the number of positive autoantibodies suggests that, in the context of clinician-diagnosed type 1 diabetes, single autoantibody-positive individuals are unlikely to represent false positives.

Our estimate of the proportion of individuals with non-autoimmune diabetes based on the T1DGRS was comparable to that in a recent study using biomarkers (C-peptide) to define type 1 diabetes rather than clinical diagnosis [34]. Foteinopoulou et al identified non-autoimmune diabetes in 11% of those with clinician-diagnosed adult-onset type 1 diabetes, comparable to the estimated 13% (147/1112) of all adults misclassified in our study [34]. The overlapping clinical features of type 1 diabetes and type 2 diabetes [35] and the relative rarity of type 1 diabetes (5% of all those with diabetes) are the major factors underlying misclassification in adults. The presence of non-autoimmune diabetes is supported by the high proportion of autoantibody-negative adults with the HLA DR15-DQ6 genotype (15%), which is highly protective against type 1 diabetes. The higher BMI, higher percentage of men and lower prevalence of other autoimmune diseases also point towards the presence of type 2 diabetes as a major contributor to non-autoimmune diabetes in this group. It is well reported that a family history of diabetes is more common in type 2 diabetes than in type 1 diabetes [36–38]; therefore, the higher prevalence of a family history of diabetes in the autoantibody-negative group (despite a reduced T1DGRS) also supports the inadvertent inclusion of individuals with type 2 diabetes in this group. These individuals with possible type 2 diabetes are likely to represent a very small fraction of the total type 2 diabetes population and are atypical. This is highlighted by the high rate of diabetic ketoacidosis in the autoantibody-negative adults, possibly reflecting either previously proposed glucotoxicity-induced transient insulin deficiency [39] or the severe insulin-deficient subtype of type 2 diabetes proposed by Ahlqvist et al [40], who observed that 25% of this cluster had diabetic ketoacidosis at presentation. It would be interesting to analyse the type 2 diabetes genetic risk in these patients and carry out long-term follow-up with physiological studies of insulin secretion to better understand the aetiology of diabetic ketoacidosis in autoantibody-negative individuals.

Genetic testing identified only seven adults with monogenic diabetes, supporting the presence of misclassification but not explaining most of the estimated misclassified individuals. The finding of individuals with confirmed monogenic diabetes and possible type 2 diabetes among autoantibody-negative adults would support autoantibody testing in all those with suspected adult-onset type 1 diabetes. Future analysis of atypical non-autoimmune individuals presenting clinically with features suggestive of insulin deficiency (diabetic ketoacidosis) would be helpful to determine the clinical benefit of identifying these individuals. Studies evaluating patients presenting with diabetic ketoacidosis showed that a substantial proportion had retained C-peptide at follow-up and were able to successfully stop insulin therapy [41, 42]. Therefore, we suggest that it is reasonable to serially evaluate C-peptide in autoantibody-negative individuals to identify those with retained endogenous insulin secretion who may benefit from non-insulin therapies [34, 43]. Genetic testing for monogenic diabetes should be considered when the probability of monogenic diabetes is high, for example when there is a strong family history or a higher probability of monogenic diabetes based on a clinical model [37].

Our observation of a higher proportion of individuals with autoantibody-negative type 1 diabetes among adults with an intermediate genetic predisposition to type 1 diabetes could be explained by the presence of a discrete form of autoimmune diabetes associated with a lower genetic risk in which the autoimmunity is not captured by the three autoantibodies measured. However, the differences in clinical features between autoantibody-negative and autoantibody-positive adults but not children, the presence of monogenic diabetes in the autoantibody-negative group, the similar T1DGRS by autoantibody status in childhood-onset type 1 diabetes and the lack of association of T1DGRS with number of positive autoantibodies do not completely support this hypothesis. The fact that in autoantibody-negative adults a low genetic predisposition to type 1 diabetes was associated with a higher BMI and older age at diagnosis further suggests that the presence of a discrete type 1 diabetes subgroup does not explain our study results. This finding is supported by previous literature showing that in autoantibody-negative young adults with diabetes the absence of HLA-DQ risk genotypes identifies a cohort with markedly different clinical features and C-peptide levels from those with HLA-DQ risk [44]. Further detailed genetic analysis, immunophenotyping and clinical studies are needed to investigate the possible presence of a discrete form of diabetes.

Our study has several limitations. We did not have information on C-peptide at diagnosis but it has limited utility close to diagnosis and thus is unlikely to have changed the results [45]. We used a 30 variant T1DGRS in our study rather than the recently published 67 variant T1DGRS [46]. The 67 variant T1DGRS has only modestly improved discriminatory power and therefore this is unlikely to have significantly altered the results [31]. The weights for the T1DGRS were derived from genome-wide association studies in children and adolescents rather than older adults, as no genome-wide association weights are available for adults only. While this may have affected the performance of the score in adults with type 1 diabetes [47], because of the within-age group analysis strategy of our study it would not have impacted our results [31]. Our analysis was limited to White Europeans because of the lack of weights for the T1DGRS in non-European ancestry and a lack of power for meaningful analysis in non-European ancestry in our cohort (59 South Asian and 45 African participants). Insulin autoantibodies (IAA) were not analysed in this study because participants were treated with exogenous insulin for a median of 3 months, making this analysis unreliable. In previous studies in which GADA, IA2A, ZNT8A and IAA were measured concurrently, only 2% of participants were positive for only IAA [48, 49]; therefore, the absence of IAA measurement is unlikely to have significantly affected our results. Furthermore, IAA prevalence is known to decrease with increasing age at diagnosis, so undetected IAA would not have explained the increasing prevalence of negative autoantibodies with age [48]. It is possible that autoantibody status may change over time [50, 51], but this is unlikely to explain the reduction in genetic predisposition to type 1 diabetes in autoantibody-negative adults given the lack of difference in autoantibody-positive and antibody-negative children and the lack of an interaction between T1DGRS and number of positive autoantibodies.

In summary, our detailed immunogenetic study of a large group of individuals with clinician-diagnosed adult-onset type 1 diabetes highlights the possible causes of the apparently lower prevalence of positive autoantibodies in adults. Misclassification of type 1 diabetes is the most likely explanation for this observation. The high rate of misclassification in adults who are clinically suspected of having type 1 diabetes strongly supports the routine measurement of autoantibodies in all individuals and not just when there is diagnostic uncertainty.

## Supplementary information

ESM 1 (PDF 924 kb)

## Data Availability

ADDRESS-2 data access is available via a management committee [21].

## Abbreviations

GADA: GAD autoantibodies
IA2A: Insulinoma-2 antigen autoantibodies
IAA: Insulin autoantibodies
T1DGRS: Type 1 diabetes genetic risk score
ZnT8A: Zinc transporter-8 autoantibodies

## References

[1] Thomas NJ, Jones SE, Weedon MN, Shields BM, Oram RA, Hattersley AT (2018). Frequency and phenotype of type 1 diabetes in the first six decades of life: a cross-sectional, genetically stratified survival analysis from UK Biobank. Lancet Diabetes Endocrinol.

[2] Bravis V, Kaur A, Walkey HC (2018). Relationship between islet autoantibody status and the clinical characteristics of children and adults with incident type 1 diabetes in a UK cohort. BMJ Open.

[3] Marcus C (2018) Better diabetes diagnoses in Sweden [article in Swedish]. Lakartidningen 115:EXDS 29509210

[4] Notkins AL, Lernmark A (2001). Autoimmune type 1 diabetes: resolved and unresolved issues. J Clin Investig.

[5] Tridgell DM, Spiekerman C, Wang RS, Greenbaum CJ (2011). Interaction of onset and duration of diabetes on the percent of GAD and IA-2 antibody-positive subjects in the type 1 diabetes genetics consortium database. Diabetes Care.

[6] Graham J, Hagopian WA, Kockum I (2002). Genetic effects on age-dependent onset and islet cell autoantibody markers in type 1 diabetes. Diabetes.

[7] Rogowicz-Frontczak A, Pilacinski S, Wyka K, Wierusz-Wysocka B, Zozulinska-Ziolkiewicz D (2018). Zinc transporter 8 autoantibodies (ZnT8-ab) are associated with higher prevalence of multiple diabetes-related autoantibodies in adults with type 1 diabetes. Diabetes Res Clin Pract.

[8] Vermeulen I, Weets I, Asanghanwa M (2011). Contribution of antibodies against IA-2beta and zinc transporter 8 to classification of diabetes diagnosed under 40 years of age. Diabetes Care.

[9] Sabbah E, Savola K, Ebeling T (2000). Genetic, autoimmune, and clinical characteristics of childhood- and adult-onset type 1 diabetes. Diabetes Care.

[10] Lernmark A (2021). Etiology of autoimmune islet disease: timing is everything. Diabetes.

[11] Ilonen J, Laine AP, Kiviniemi M (2022). Associations between deduced first islet specific autoantibody with sex, age at diagnosis and genetic risk factors in young children with type 1 diabetes. Pediatr Diabetes.

[12] Mikk ML, Pfeiffer S, Kiviniemi M (2020). HLA-DR-DQ haplotypes and specificity of the initial autoantibody in islet specific autoimmunity. Pediatr Diabetes.

[13] Hagopian WA, Sanjeevi CB, Kockum I (1995). Glutamate decarboxylase-, insulin-, and islet cell-antibodies and HLA typing to detect diabetes in a general population-based study of Swedish children. J Clin Invest.

[14] Sanjeevi CB, Hagopian WA, Landin-Olsson M (1998). Association between autoantibody markers and subtypes of DR4 and DR4-DQ in Swedish children with insulin-dependent diabetes reveals closer association of tyrosine pyrophosphatase autoimmunity with DR4 than DQ8. Tissue Antigens.

[15] Noble JA (2015). Immunogenetics of type 1 diabetes: a comprehensive review. J Autoimmun.

[16] Grant SFA, Wells AD, Rich SS (2020). Next steps in the identification of gene targets for type 1 diabetes. Diabetologia.

[17] Oram RA, Patel K, Hill A (2015). A type 1 diabetes genetic risk score can aid discrimination between type 1 and type 2 diabetes in young adults. Diabetes Care.

[18] Patel KA, Oram RA, Flanagan SE (2016). Type 1 diabetes genetic risk score: a novel tool to discriminate monogenic and type 1 diabetes. Diabetes.

[19] Howson JM, Rosinger S, Smyth DJ, Boehm BO, Todd JA, Group A-ES (2011). Genetic analysis of adult-onset autoimmune diabetes. Diabetes.

[20] McKeigue PM, Spiliopoulou A, McGurnaghan S (2019). Persistent C-peptide secretion in type 1 diabetes and its relationship to the genetic architecture of diabetes. BMC Med.

[21] Walkey HC, Kaur A, Bravis V (2017). Rationale and protocol for the After Diabetes Diagnosis REsearch Support System (ADDRESS): an incident and high risk type 1 diabetes UK cohort study. BMJ Open.

[22] American Diabetes Association (2021). 2. Classification and diagnosis of diabetes: Standards of medical care in diabetes—2021. Diabetes Care.

[23] Leslie RD, Lernmark A (2018). Genetic risk scores in adult-onset type 1 diabetes. Lancet Diabetes Endocrinol.

[24] Bonifacio E, Yu L, Williams AK (2010). Harmonization of glutamic acid decarboxylase and islet antigen-2 autoantibody assays for national institute of diabetes and digestive and kidney diseases consortia. J Clin Endocrinol Metab.

[25] Long AE, Gooneratne AT, Rokni S, Williams AJ, Bingley PJ (2012). The role of autoantibodies to zinc transporter 8 in prediction of type 1 diabetes in relatives: lessons from the European Nicotinamide Diabetes Intervention Trial (ENDIT) cohort. J Clin Endocrinol Metab.

[26] Rich SS, Concannon P, Erlich H (2006). The type 1 diabetes genetics consortium. Ann N Y Acad Sci.

[27] Ellard S, Lango Allen H, De Franco E (2013). Improved genetic testing for monogenic diabetes using targeted next-generation sequencing. Diabetologia.

[28] de Onis M, Onyango AW, Borghi E, Siyam A, Nishida C, Siekmann J (2007). Development of a WHO growth reference for school-aged children and adolescents. Bull World Health Organ.

[29] Wellcome Trust Case Control Consortium (2007). Genome-wide association study of 14,000 cases of seven common diseases and 3,000 shared controls. Nature.

[30] Mishra R, Chesi A, Cousminer DL (2017). Relative contribution of type 1 and type 2 diabetes loci to the genetic etiology of adult-onset, non-insulin-requiring autoimmune diabetes. BMC Med.

[31] Evans BD, Słowiński P, Hattersley AT (2021). Estimating disease prevalence in large datasets using genetic risk scores. Nat Commun.

[32] McDonald TJ, Colclough K, Brown R (2011). Islet autoantibodies can discriminate maturity-onset diabetes of the young (MODY) from type 1 diabetes. Diabet Med.

[33] Thomas NJ, Lynam AL, Hill AV (2019). Type 1 diabetes defined by severe insulin deficiency occurs after 30 years of age and is commonly treated as type 2 diabetes. Diabetologia.

[34] Foteinopoulou E, Clarke CAL, Pattenden RJ (2021). Impact of routine clinic measurement of serum C-peptide in people with a clinician-diagnosis of type 1 diabetes. Diabetic Med.

[35] Shields BM, Peters JL, Cooper C (2015). Can clinical features be used to differentiate type 1 from type 2 diabetes? A systematic review of the literature. BMJ Open.

[36] Meigs JB, Cupples LA, Wilson PW (2000). Parental transmission of type 2 diabetes: the Framingham Offspring Study. Diabetes.

[37] Shields BM, McDonald TJ, Ellard S, Campbell MJ, Hyde C, Hattersley AT (2012). The development and validation of a clinical prediction model to determine the probability of MODY in patients with young-onset diabetes. Diabetologia.

[38] Svensson E, Berencsi K, Sander S (2016). Association of parental history of type 2 diabetes with age, lifestyle, anthropometric factors, and clinical severity at type 2 diabetes diagnosis: results from the DD2 study. Diabetes Metab Res Rev.

[39] Puttanna A, Padinjakara R (2014). Diabetic ketoacidosis in type 2 diabetes mellitus. Practical Diabetes.

[40] Ahlqvist E, Storm P, Karajamaki A (2018). Novel subgroups of adult-onset diabetes and their association with outcomes: a data-driven cluster analysis of six variables. Lancet Diabetes Endocrinol.

[41] Maldonado M, Hampe CS, Gaur LK (2003). Ketosis-prone diabetes: dissection of a heterogeneous syndrome using an immunogenetic and beta-cell functional classification, prospective analysis, and clinical outcomes. J Clin Endocrinol Metab.

[42] Seok H, Jung CH, Kim SW (2013). Clinical characteristics and insulin independence of Koreans with new-onset type 2 diabetes presenting with diabetic ketoacidosis. Diabetes Metab Res Rev.

[43] Thanabalasingham G, Owen KR (2011). Diagnosis and management of maturity onset diabetes of the young (MODY). BMJ.

[44] Weets I, Siraux V, Daubresse JC (2002). Relation between disease phenotype and HLA-DQ genotype in diabetic patients diagnosed in early adulthood. J Clin Endocrinol Metab.

[45] Jones AG, Hattersley AT (2013). The clinical utility of C-peptide measurement in the care of patients with diabetes. Diabetic Med.

[46] Sharp SA, Rich SS, Wood AR (2019). Development and standardization of an improved type 1 diabetes genetic risk score for use in newborn screening and incident diagnosis. Diabetes Care.

[47] Battaglia M, Ahmed S, Anderson MS (2020). Introducing the endotype concept to address the challenge of disease heterogeneity in type 1 diabetes. Diabetes Care.

[48] Wenzlau JM, Juhl K, Yu L (2007). The cation efflux transporter ZnT8 (Slc30A8) is a major autoantigen in human type 1 diabetes. Proc Natl Acad Sci U S A.

[49] Redondo MJ, Sosenko J, Libman I (2020). Single islet autoantibody at diagnosis of clinical type 1 diabetes is associated with older age and insulin resistance. J Clin Endocrinol Metab.

[50] Savola K, Sabbah E, Kulmala P, Vahasalo P, Ilonen J, Knip M (1998). Autoantibodies associated with type I diabetes mellitus persist after diagnosis in children. Diabetologia.

[51] Long AE, George G, Williams CL (2021). Persistence of islet autoantibodies after diagnosis in type 1 diabetes. Diabet Med.

