# Supplementary material for: The relationship between islet autoantibody status and the genetic risk of type 1 diabetes in adult-onset type 1 diabetes
Source: Diabetologia. 2022 Nov 10;66(2):310–20. doi: 10.1007/s00125-022-05823-1 (PMC9807542; doi:10.1007/s00125-022-05823-1)
Supplement: Supplementary file 1 — (PDF 924 kb) [file 125_2022_5823_MOESM1_ESM.pdf]

ESM

ESM methods

Participant characterization

Diabetic Ketoacidosis definition

Diabetic ketoacidosis at diagnosis was recorded if assigned by the local clinical team, or if all the following were documented in the clinical records: glucose >11mmol/L; blood ketone bodies >3mmol/L or urine ketones levels positive ++; pH <7.3; and/or bicarbonate <15 mmol/L.

Definition of another autoimmune condition

Presence of another autoimmune condition was recorded if reporting one or more of: autoimmune hyperthyroidism, autoimmune hypothyroidism, coeliac disease, Addison's disease or vitiligo.

Genetic analysis

Generation of a type 1 diabetes genetic risk score

We generated a T1DGRS, a measure of an individual's genetic susceptibility to T1D, from 30 common genetic variants associated with T1D as previously described (1, 2). These variants were directly genotyped by LGC genomics (Middlesex, UK) as previously described (1). The T1DGRS was generated by summing the effective allele dosage of each variant multiplied by the natural log (ln) of the odds ratio (1, 2). We also generated separate T1DGRS based on only HLA (6 SNPS, HLA-T1DGRS) and non-HLA (24 SNPS, non-HLA-T1DGRS) variants for comparison.

HLA genotypes

HLA genotypes were imputed based on validated tagging variants as previously described. DR3-DQ2 (*DRB1*03:01-DQA1*05:01-DQB1*02:01*) and DR4-DQ8 (*DRB1*04-DQA1*03-DQB1*03:02*) were tagged by rs2187668 and rs7454108, respectively with the protective HLA DR15-DQ6 (*DRB1*15-DQA1*01-DQB1*06*) tagged by rs3129889 as described (1-3).

Testing for monogenic forms of diabetes

The coding regions and 50 nucleotides of flanking intronic sequence of 28 known monogenic diabetes genes (see ESM for full list) and the mitochondrial DNA mutation m.3243A>G were analyzed using the Agilent SureSelect custom capture library and an Illumina NetSeq 500 sequencing platform according to the methodology described by Ellard *et al* (4). Interpretation and classification

of sequence variants was undertaken based on the American College of Medical Genetics and Genomics (ACMG) guidelines (5). Only variants classified as likely pathogenic (class 4) or pathogenic (class 5) were included in the study.

Monogenic diabetes genes tested

GCK, *HNF1A*, *HNF4A*, *HNF1B*, *INS*, *CEL*, *PDX1*, *PAX4*, *BLK*, *KLF11*, *KCNJ11*, *ABCC8*, *PTF1A*, *NEUROD1*, *NEUROG3*, *RFX6*, *EIF2AK3*, *FOXP3*, *GLIS3*, *SLC19A2*, *SLC2A2*, *IER3IP1*, *ZFP57*, *WFS1*,*GATA6*, *GATA4,* PPARG LMNA *and* m.3243,

Control cohort of non-autoimmune diabetes cases

We used well characterized cases of T2D of European ancestry from the Wellcome Trust Case Control Consortium (WTCCCC) cohort (n=1924) as a representative cohort for non-autoimmune diabetes. All of these cases were negative for GADA, lacked family history of T1D and were not insulin treated for 1 year from diagnosis (6). T1DGRS generation on this cohort is previously described (1, 2). T1DGRS of all non-autoimmune diabetes groups is similar whether it is non-diabetes controls or those with non-autoimmune diabetes irrespective of the underlying cause (i.e. T2D, monogenetic or secondary diabetes) (1, 2).

ESM Table 1: Clinical features of clinically diagnosed type 1 diabetes, data shown separately for adults and children. Data are mean (SD) or n (%).* Body mass index (BMI) was age adjusted using WHO (2007) reference data.

		All<br>N=1814	<18<br>N=702	≥18<br>N=1112
**Age at diagnosis (years)**		25 (14)	12 (3)	33 (12)
**Sex**	**Male**	1088 (60%)	400 (57%)	688 (62%)
	**Female**	726 (40%)	302 (43%)	424 (38%)
**Diabetes duration (weeks)**		11 (7)	13 (7)	11 (7)
**Baseline HbA1c (mmol/mol)**		87 (33)	77 (31)	93 (33)
**Baseline HbA1c (%)**		10.1 (5.2)	9.2 (5.0)	10.7 (5.2)
**BMI (kg/m2)***		24 (5)	23 (4)	25 (6)
**≥1 autoantibody positive**		1536 (85%)	642 (91%)	894 (80%)
**GADA**		1268 (70%)	463 (66%)	805 (72%)
**IA2A**		1034 (57%)	517 (74%)	517 (46%)
**ZNT8A**		906 (50%)	449 (64%)	457 (41%)
**Autoantibody negative**		278 (15%)	60 (9%)	218 (20%)
**1 autoantibody Positive**		434 (24%)	125 (18%)	309 (28%)
**2 autoantibody Positive**		532 (29%)	247 (35%)	285 (26%)
**3 autoantibody Positive**		570 (31%)	270 (38%)	300 (27%)
**Diabetic Ketoacidosis**		737 (41%)	277 (39%)	460 (41%)
**Hospitalised at admission**		1345 (74%)	656 (93%)	689 (62%)
**Weight loss at diagnosis**		1535 (85%)	576 (82%)	959 (86%)
**Polyuria or polydipsia**		1715 (95%)	677 (96%)	1038 (93%)
**Another autoimmune disease**		122 (7%)	26 (4%)	96 (9%)
**Parent with diabetes**		322 (18%)	79 (11%)	243 (22%)

ESM Table 2: Clinical features of clinically diagnosed adult onset type 1 diabetes split by median age of diagnosis. Data are mean (SD) or n (%).

		18-30.95<br>n=556	>30.95<br>n=556	p
**Age at diagnosis (years)**		24 (4)	43 (9)	<0.0001
**Sex**	**Male**	343 (62%)	345 (62%)	0.9
	**Female**	213 (38%)	211 (38%)	0.9
**Diabetes duration (weeks)**		10 (7)	11 (7)	0.02
**Baseline HbA1c (mmol/mol)**		91 (33)	94 (32)	0.2
**Baseline HbA1c (%)**		10.5 (5.2)	10.8 (5.1)	0.2
**BMI (kg/m2)**		24 (5)	26 (6)	<0.001
**≥1 autoantibody positive**		487 (88%)	407 (73%)	<0.0001
**GADA**		420 (76%)	385 (69%)	0.02
**IA2A**		314 (56%)	203 (37%)	<0.0001
**ZNT8A**		273 (49%)	184 (33%)	<0.0001
**Autoantibody negative**		69 (12%)	149 (27%)	<0.0001
**1 autoantibody Positive**		147 (26%)	162 (29%)	<0.0001
**2 autoantibody Positive**		160 (29%)	125 (22%)	<0.0001
**3 autoantibody Positive**		180 (32%)	120 (22%)	<0.0001
**Diabetic Ketoacidosis**		234 (42%)	226 (41%)	0.6
**Hospitalised at admission**		369 (66%)	320 (58%)	<0.01
**Weight loss at diagnosis**		467 (84%)	492 (88%)	0.03
**Polyuria or polydipsia**		520 (94%)	518 (93%)	0.8
**Another autoimmune disease**		26 (5%)	70 (13%)	<0.0001
**Parent with diabetes**		102 (18%)	141 (25%)	<0.01

ESM Table 3: Genetic profile of clinically diagnosed type 1 diabetes split by islet autoantibody status for adults and children. Data are mean (SD) or n (%).

	Children (<18) autoantibody positive n=642	Children (<18) autoantibody negative n=60	p	Adult (≥18) autoantibody positive n=894	Adult (≥18) autoantibody negative n=218	p
T1DGRS	0.277 (0.026)	0.274 (0.034)	0.4	0.271 (0.026)	0.243 (0.036)	<0.0001
Non-HLA T1DGRS	0.129 (0.014)	0.127 (0.014)	0.4	0.128 (0.014)	0.123 (0.016)	<0.0001
HLA T1DGRS	1.200 (0.172)	1.188 (0.212)	0.6	1.164 (0.181)	0.988 (0.229)	<0.0001
X/X	85 (13%)	10 (17%)	0.2	148 (17%)	82 (38%)	<0.0001
DR3-DQ2/X or DR4-DQ8/X	366 (57%)	28 (47%)		552 (62%)	91 (42%)	
DR3-DQ2/DR4-DQ8	181 (28%)	20 (33%)		170 (19%)	12 (6%)	
DQ6-DR15	10 (2%)	2 (3%)		24 (3%)	33 (15%)	

ESM Table 4. Clinical features of clinically diagnosed adult-onset type 1 diabetes split by median age of onset and autoantibody status. Data are mean (SD) or n (%).

		Diagnosed 18-31 years of age, autoantibody positive, n=487	Diagnosed 18-31 years of age, autoantibody negative n=69	p	Diagnosed >31 years of age, autoantibody positive, n=407	Diagnosed >31 years of age, autoantibody negative n=149	p
**At diagnosis**							
Age at diagnosis (years)		24 (4)	25 (3)	0.2	43 (9)	44 (10)	0.3
Sex	Male	296 (61%)	47 (68%)	0.2	228 (56%)	117 (79%)	<0.0001
	Female	191 (39%)	22 (32%)	0.2	179 (44%)	32 (21%)	<0.0001
Diabetic Ketoacidosis		205 (42%)	29 (42%)	0.99	172 (42%)	54 (36%)	0.2
Weight loss		412 (85%)	55 (80%)	0.3	361 (89%)	131 (88%)	0.8
Polyuria or polydipsia		458 (94%)	62 (90%)	0.2	381 (94%)	137 (92%)	0.5
Hospitalised at admission		325 (67%)	44 (64%)	0.6	234 (57%)	86 (58%)	0.96
**At Recruitment**							
Diabetes duration (weeks)		10 (7)	10 (7)	0.9	11 (7)	11 (7)	0.3
HbA1c (mmol/mol)		90 (33)	98 (37)	0.08	92 (30)	101 (35)	0.004
BMI (kg/m2)		24 (5)	26 (7)	0.01	25 (5)	27 (7)	0.001
On concurrent oral hypoglycaemic agent		6 (1%)	9 (13%)	<0.0001	35 (9%)	14 (9%)	0.8
Another autoimmune disease		24 (5)	2 (3%)	0.5	68 (17%)	2 (1%)	<0.0001
Parent with Diabetes		75 (15%)	27 (39%)	<0.0001	96 (24%)	45 (30%)	0.1
**Genetics**							
T1DGRS		0.271 (0.026)	0.252 (0.039)	<0.0001	0.270 (0.027)	0.238 (0.034)	<0.0001
Non-HLA T1DGRS		0.128 (0.015)	0.127 (0.016)	0.6	0.128 (0.014)	0.122 (0.016)	<0.0001
HLA T1DGRS		1.168 (0.169)	1.034 (0.253)	<0.0001	1.158 (0.195)	0.966 (0.214)	<0.0001
X/X		82 (17%)	23 (33%)	<0.0001	66 (16%)	59 (40%)	<0.0001
DR3-DQ2 or DR4-DQ8 containing , without DQ6-DR15		303 (62%)	30 (43%)		249 (61%)	61 (41%)	
DR3-DQ2/DR4-DQ8		95 (20%)	8 (12%)		75 (18%)	4 (3%)	
DQ6-DR15		7 (1%)	8 (12%)		17 (4%)	25 (17%)	

ESM Table 5. Individual frequency of non-HLA variants by autoantibody status

		autoantibody positive, Copies of each SNP			autoantibody negative, Copies of each SNP			
SNP	Gene	0	1	2	0	1	2	p
rs2476601	PTPN22	605 (68%)	258 (29%)	31 (3%)	163 (75%)	49 (22%)	6 (3%)	0.1
rs689	INS	29 (3%)	231 (26%)	630 (71%)	16 (7%)	78 (36%)	123 (57%)	<0.0001
rs12722495	IL2RA	12 (1%)	163 (18%)	718 (80%)	3 (1%)	40 (18%)	175 (80%)	0.99
rs2292239	ERBB3	342 (38%)	422 (47%)	130 (15%)	86 (40%)	100 (46%)	31 (14%)	0.932
rs10509540	C10orf59	63 (7%)	318 (36%)	513 (57%)	24 (11%)	77 (35%)	117 (54%)	0.1
rs4948088	COBL	1 (0%)	62 (7%)	829 (93%)	1 (0%)	17 (8%)	200 (92%)	0.5
rs7202877		713 (80%)	171 (19%)	9 (1%)	170 (78%)	44 (20%)	2 (1%)	0.9
rs12708716	CLEC16A	86 (10%)	370 (41%)	435 (49%)	22 (10%)	95 (44%)	100 (46%)	0.8
rs3087243	CTLA4	154 (17%)	415 (46%)	323 (36%)	34 (16%)	107 (49%)	77 (35%)	0.8
rs1893217	PTPN2	556 (62%)	302 (34%)	32 (4%)	143 (66%)	68 (31%)	7 (3%)	0.7
rs11594656	IL2RA	42 (5%)	323 (36%)	526 (59%)	18 (8%)	69 (32%)	131 (60%)	0.08
rs3024505	IL10	11 (1%)	222 (25%)	659 (74%)	3 (1%)	60 (28%)	155 (71%)	0.7
rs9388489	C6orf173	256 (29%)	434 (49%)	203 (23%)	59 (27%)	108 (50%)	50 (23%)	0.9
rs1465788		49 (5%)	345 (39%)	500 (56%)	16 (7%)	84 (39%)	117 (54%)	0.6
rs1990760	IFIH1	117 (13%)	427 (48%)	343 (38%)	38 (18%)	102 (47%)	73 (34%)	0.2
rs3825932	CTSH	76 (9%)	383 (43%)	435 (49%)	22 (10%)	94 (43%)	102 (47%)	0.7
rs425105		26 (3%)	223 (25%)	640 (72%)	4 (2%)	54 (25%)	156 (72%)	0.7
rs763361	CD226	224 (25%)	430 (48%)	240 (27%)	47 (22%)	114 (53%)	56 (26%)	0.5
rs4788084	IL27	146 (16%)	406 (45%)	341 (38%)	35 (16%)	99 (46%)	84 (39%)	0.99
rs17574546		560 (63%)	284 (32%)	49 (5%)	144 (66%)	63 (29%)	10 (5%)	0.6
rs11755527	BACH2	211 (24%)	453 (51%)	228 (26%)	57 (26%)	116 (53%)	44 (20%)	0.3
rs3788013	UBSH3A	252 (28%)	462 (52%)	178 (20%)	70 (32%)	107 (49%)	41 (19%)	0.5
rs2069762	IL2	78 (9%)	356 (40%)	457 (51%)	18 (8%)	101 (47%)	99 (46%)	0.2
rs2281808		99 (11%)	397 (44%)	397 (44%)	30 (14%)	101 (47%)	87 (40%)	0.4
rs5753037		328 (37%)	427 (48%)	137 (15%)	59 (27%)	121 (56%)	37 (17%)	0.03

ESM Table 6: Clinical features of clinically diagnosed childhood-onset type 1 diabetes by autoantibody status. Data are mean (SD) or n (%). * Body mass index (BMI) was age adjusted using WHO (2007) reference data.

		Autoantibody positive n=642	Autoantibody negative n=60	p
**At diagnosis**				
Age at diagnosis (years)		12 (3)	11 (4)	0.005
Sex	Male	364 (57%)	36 (60%)	0.6
	Female	278 (43%)	24 (40%)	0.6
Diabetic Ketoacidosis		259 (40%)	18 (30%)	0.1
Weight loss		523 (81%)	53 (88%)	0.2
Polyuria or polydipsia		619 (96%)	58 (97%)	0.9
Hospitalised at admission		599 (93%)	57 (95%)	0.6
**At Recruitment**				
Diabetes duration (weeks)		13 (7)	13 (7)	0.9
HbA1c (mmol/mol)		78 (31)	75 (28)	0.6
HbA1c (%)		9.3 (5.0)	9.0 (4.7)	0.6
BMI (kg/m2)*		23 (4)	23 (3)	0.7
On concurrent oral hypoglycaemic agent		0 (0%)	0 (0%)	n/a
Another autoimmune disease		24 (4%)	2 (3%)	0.9
Parent with diabetes		68 (11%)	11 (18%)	0.07

ESM Table 7: Characteristics of islet autoantibody positive adult-onset type 1 diabetes split by number of positive islet autoantibodies. Data are mean (SD) or n (%).

		One autoantibody positive n=309	Two autoantibody positive n=285	Three autoantibody positive n=300	p
**At diagnosis**					
Age at diagnosis (years)		34 (12)	32 (11)	31 (12)	0.001
Sex	Male<br>Female	185 (60%)<br>124 (40%)	177 (62%)<br>108 (38%)	162 (54%)<br>138 (46%)	0.1<br>0.1
Diabetic Ketoacidosis		122 (39%)	111 (39%)	144 (48%)	0.03
Weight loss		263 (85%)	250 (88%)	260 (87%)	0.6
Polyuria or polydipsia		288 (93%)	269 (94%)	282 (94%)	0.7
Hospitalised at admission		192 (62%)	168 (59%)	199 (66%)	0.3
**At Recruitment**					
Diabetes duration (weeks)		11 (7)	11 (7)	11 (7)	0.95
HbA1c (mmol/mol)		91 (32)	91 (32)	90 (32)	0.8
HbA1c (%)		10.5 (5.1)	10.5 (5.1)	10.4 (5.1)	0.8
BMI (kg/m2)		25 (6)	24 (4)	25 (5)	0.98
T1DGRS		0.270 (0.027)	0.269 (0.027)	0.273 (0.025)	0.2
On concurrent oral hypoglycaemic agent		11 (4%)	18 (6%)	12 (4%)	0.8
Another autoimmune disease		30 (10%)	27 (9%)	35 (12%)	0.4
Parent with diabetes		64 (21%)	55 (19%)	52 (17%)	0.3

ESM Table 8: Characteristics of islet autoantibody positive and negative adult-onset type 1 diabetes split by the median of adult onset type 1 diabetes T1DGRS. Data are mean (SD) or n (%).

		Autoantibody positive			Autoantibody negative		
		**Low T1DGRS, n=394**	**High T1DGRS n=500**	**Low vs High p**	**Low T1DGRS, n=162**	**High T1DGRS n=56**	**Low vs High p**
**At diagnosis**							
Age at diagnosis (years)		33 (12)	32 (11)	0.2	39 (11)	34 (14)	0.02
Sex	Male<br>Female	231 (59%)<br>163 (41%)	293 (59%)<br>207 (41%)	0.99<br>0.99	120 (74%)<br>42 (26%)	44 (79%)<br>12 (21%)	0.5<br>0.5
Diabetic Ketoacidosis		169 (43%)	208 (42%)	0.7	59 (36%)	24 (43%)	0.4
Weight loss		336 (85%)	437 (87%)	0.4	136 (84%)	50 (89%)	0.3
Polyuria or polydipsia		370 (94%)	469 (94%)	0.95	146 (90%)	53 (95%)	0.3
Hospitalised at admission		254 (64%)	305 (61%)	0.3	98 (60%)	32 (57%)	0.7
**At Recruitment**							
Diabetes duration (weeks)		11 (7)	11 (7)	0.4	11 (7)	11 (7)	0.96
HbA1c (mmol/mol)		92 (31)	90 (32)	0.4	101 (36)	98 (36)	0.5
HbA1c (%)		10.6 (5.0)	10.4 (5.0)	0.4	11.4 (5.4)	11.1 (5.4)	0.5
BMI (kg/m2)		25 (5)	24 (5)	0.3	27 (7)	25 (7)	0.01
On concurrent oral hypoglycaemic agent		23 (6%)	18 (4%)	0.1	20 (12%)	5 (3%)	0.1
Another autoimmune disease		40 (10)	52 (10%)	0.9	4 (2%)	0 (0%)	0.2
Parent with Diabetes		74 (19%)	97 (19%)	0.8	53 (33%)	19 (34%)	0.9

ESM Table 9: Characteristics of monogenic cases (n=7). Data are mean (SD) or n (%).

		Diagnosed ≥18 years of age, Monogentic diabetes n=7
**Mutation**	3243>G	5 (71%)
	HNF1B	2 (29%)
**At diagnosis**		
Age at diagnosis (years)		29 (3)
Sex	Male<br>Female	6 (86%)<br>1 (14%)
Diabetic Ketoacidosis		3 (43%)
Weight loss		4 (57%)
Polyuria or polydipsia		5 (71%)
Hospitalised at admission		3 (43%)
**At Recruitment**		
Diabetes duration (weeks)		6 (4)
HbA1c (mmol/mol)		106 (31)
HbA1c (%)		11.8 (5.0)
BMI (kg/m2)		24 (4)
T1DGRS		0.218 (0.025)
On concurrent oral hypoglycaemic agent		1 (14%)
Another autoimmune disease		0 (0%)
Parent with diabetes		2 (29%)

ESM Fig. 1: Flow diagram showing case exclusions.

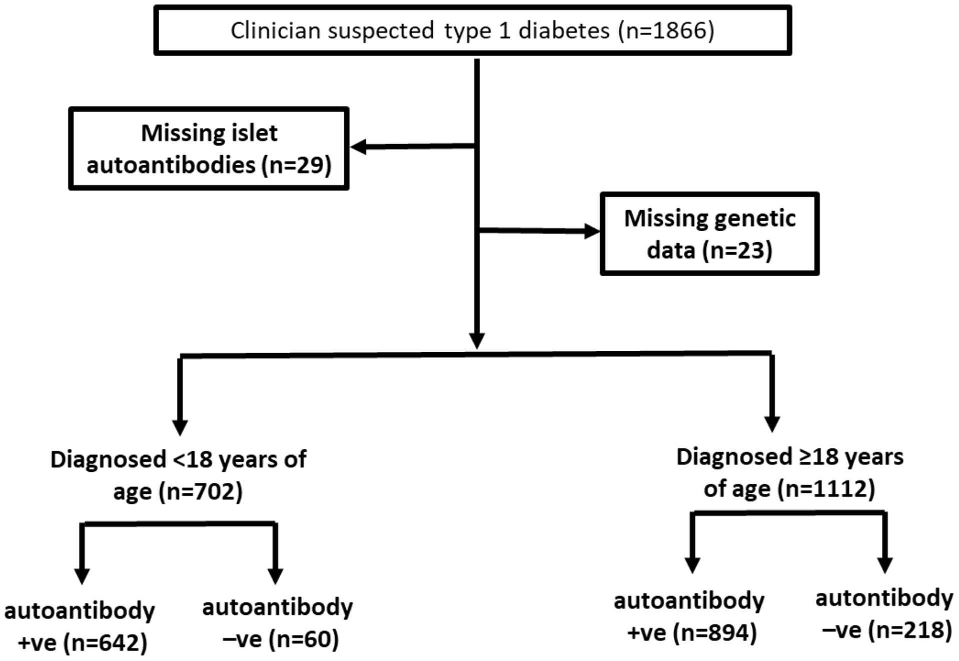

ESM Fig. 2: Type 1 diabetes genetic risk scores (T1DGRS) by islet autoantibody number in clinically diagnosed adult onset type 1 diabetes split by median age of onset and autoantibody status. T2D individuals were from the Wellcome Trust Case control consortium.

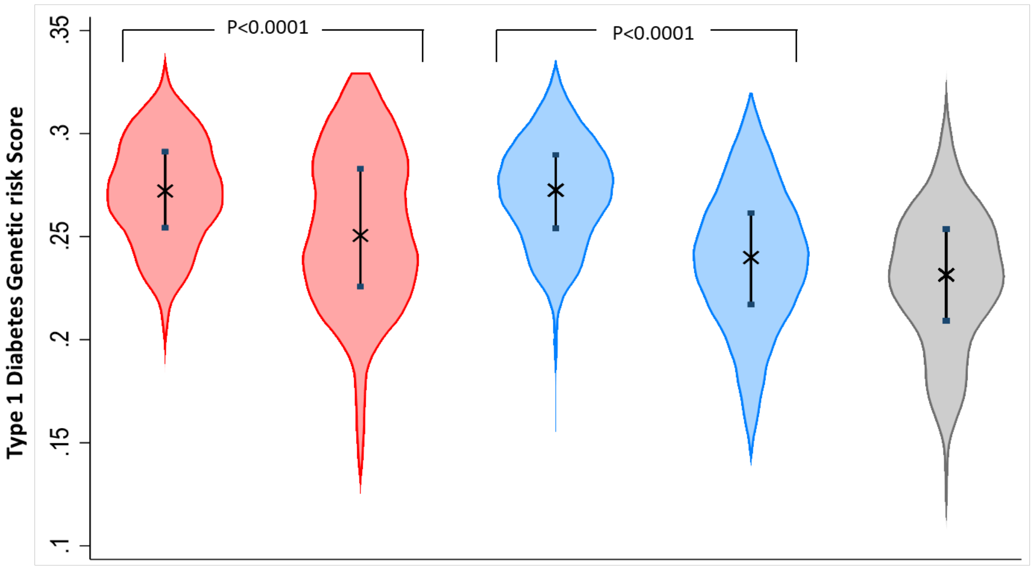

Autoantibodies	Positive	Negative	Positive	Negative	T2D
Number (n)	487	69	407	149	1924
Age at diagnosis	18 – 30.95 years		> 30.95 years		

ESM Fig. 3: The impact of ZnT8 testing on type 1 diabetes genetic risk scores (T1DGRS) in addition to GADA and IA2A testing in adults with clinically diagnosed type 1 diabetes.

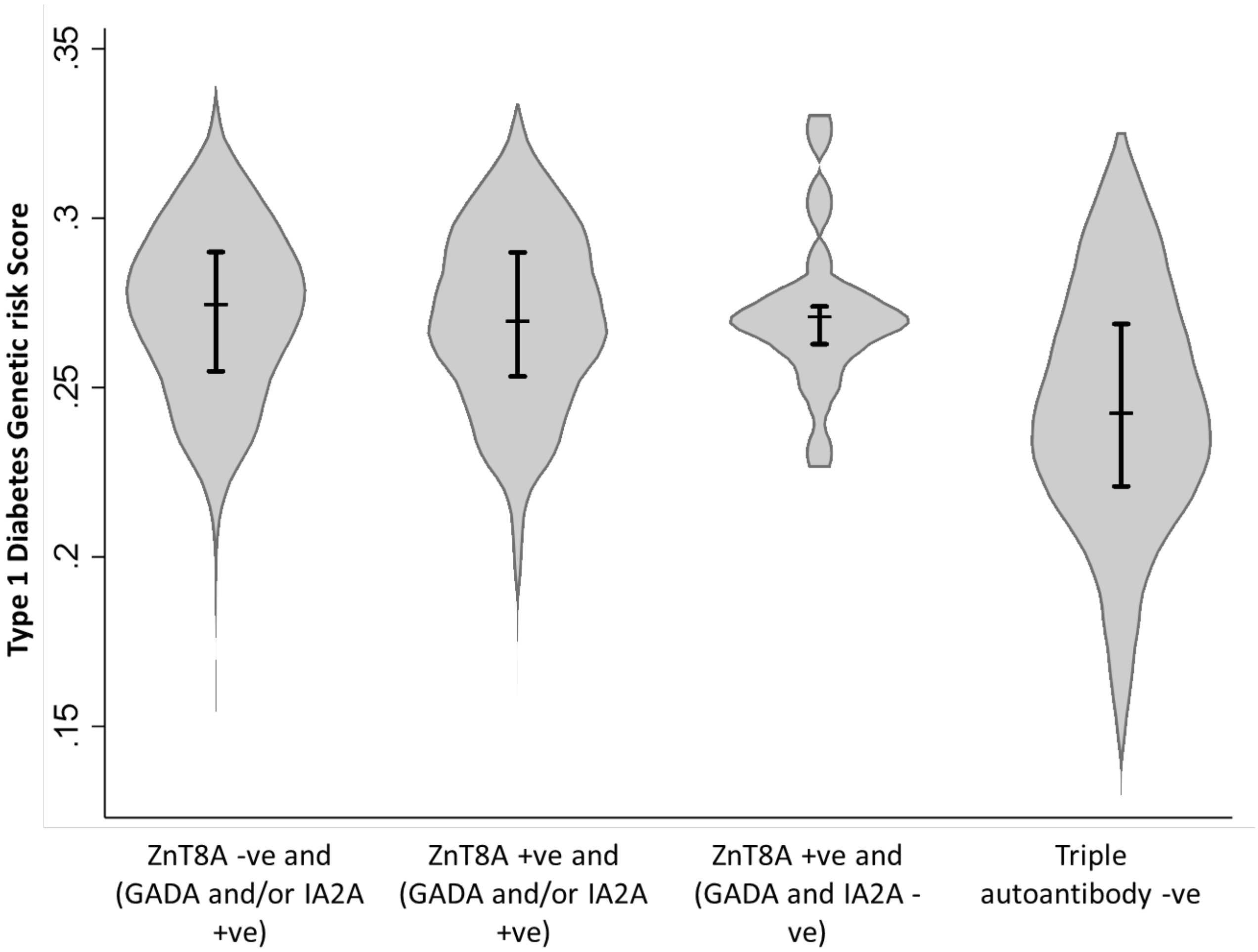

ESM Fig. 4: Type 1 diabetes genetic risk scores (T1DGRS) by islet autoantibody number in clinically diagnosed adult and childhood onset type 1 diabetes. T2D individuals were from the Wellcome Trust Case control consortium. Childhood onset diabetes were people diagnosed <18 years of age and adult onset diabetes were people diagnosed ≥18 years of age.

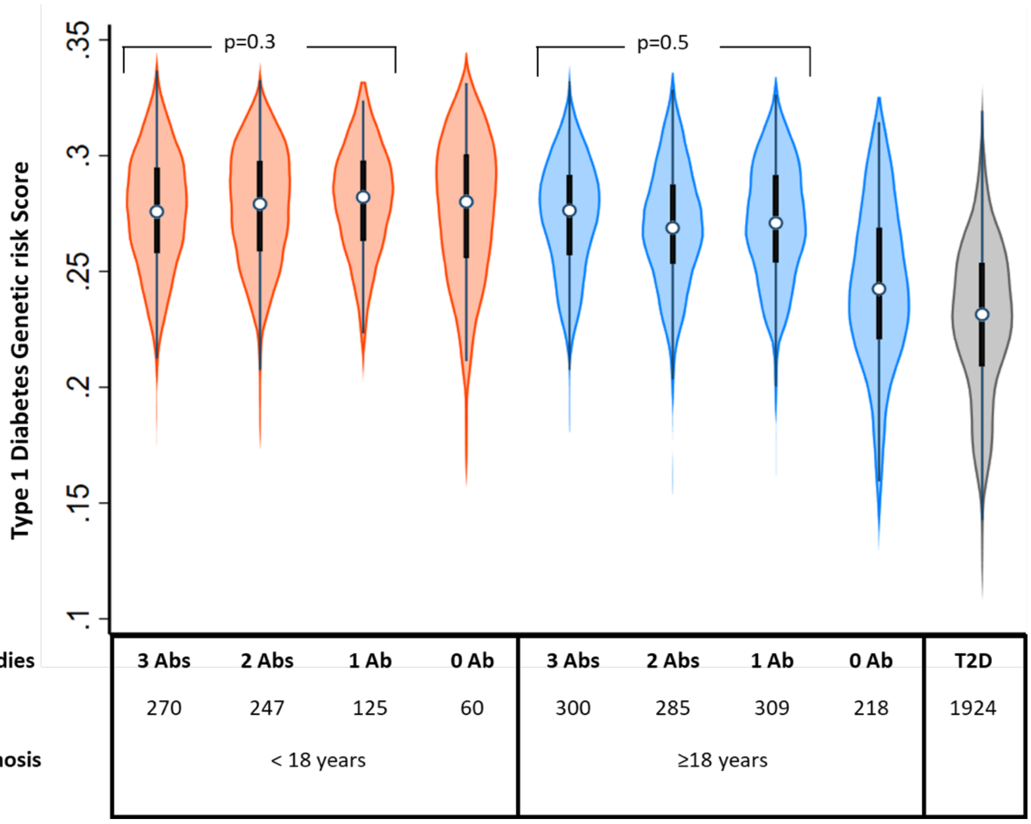

Autoantibodies	3 Abs	2 Abs	1 Ab	0 Ab	3 Abs	2 Abs	1 Ab	0 Ab	T2D
Number (n)	270	247	125	60	300	285	309	218	1924
Age at diagnosis	< 18 years				≥18 years				

ESM Fig. 5: HLA and non-HLA type 1 diabetes genetic risk score by islet autoantibody status in clinically diagnosed adult and childhood onset type 1 diabetes. T2D individuals were from the Wellcome Trust Case control consortium. Childhood onset diabetes were people diagnosed <18 years of age and adult onset diabetes were people diagnosed ≥18 years of age. Non-HLA and HLA T1DGRS were scaled for mean of 0 and standard deviation of 1 for ease of representation.

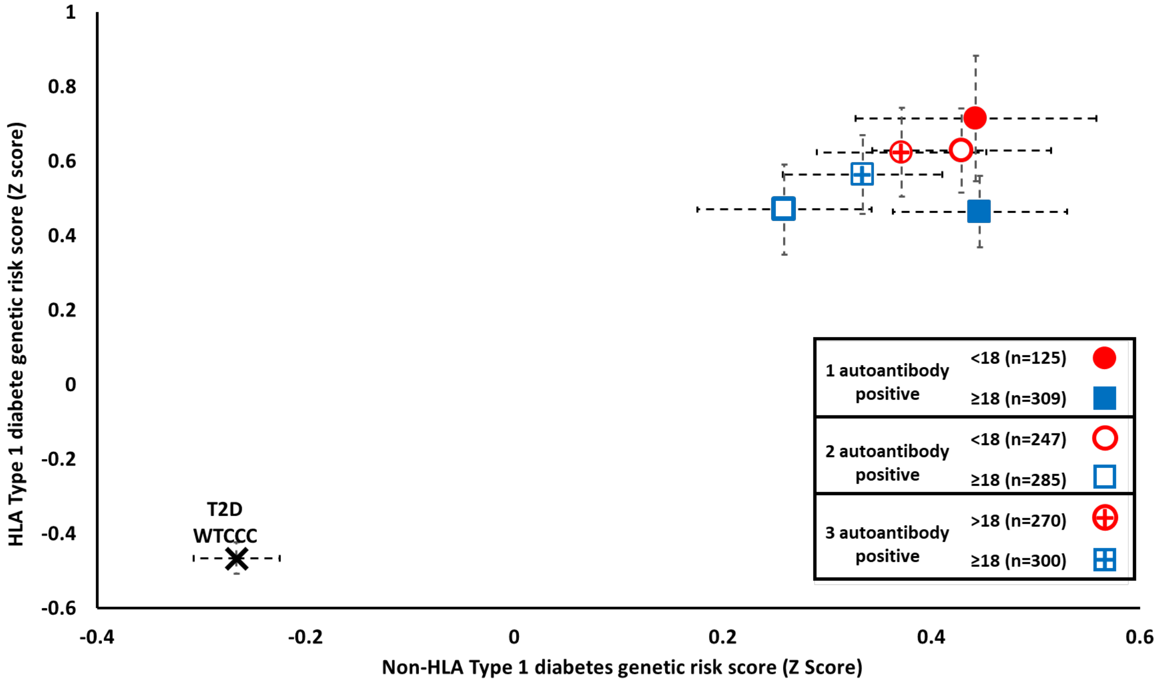

ESM references

1. Oram RA, Patel K, Hill A, Shields B, McDonald TJ, Jones A, et al. A Type 1 Diabetes Genetic Risk Score Can Aid Discrimination Between Type 1 and Type 2 Diabetes in Young Adults. Diabetes Care. 2015;39(3):337-44.
2. Patel KA, Oram RA, Flanagan SE, De Franco E, Colclough K, Shepherd M, et al. Type 1 Diabetes Genetic Risk Score: A Novel Tool to Discriminate Monogenic and Type 1 Diabetes. Diabetes. 2016;65(7):2094-9.
3. Rich SS, Concannon P, Erlich H, Julier C, Morahan G, Nerup J, et al. The Type 1 Diabetes Genetics Consortium. Annals of the New York Academy of Sciences. 2006;1079:1-8.
4. Ellard S, Lango Allen H, De Franco E, Flanagan SE, Hysenaj G, Colclough K, et al. Improved genetic testing for monogenic diabetes using targeted next-generation sequencing. Diabetologia. 2013;56(9):1958-63.
5. Richards S, Aziz N, Bale S, Bick D, Das S, Gastier-Foster J, et al. Standards and guidelines for the interpretation of sequence variants: a joint consensus recommendation of the American College of Medical Genetics and Genomics and the Association for Molecular Pathology. Genet Med. 2015;17(5):405-24.
6. Wellcome Trust Case Control C. Genome-wide association study of 14,000 cases of seven common diseases and 3,000 shared controls. Nature. 2007;447(7145):661-78.
